# Supplementary material for: Preparation of Tautomer-Pure Molecular Beams by Electrostatic Deflection
Source: J Phys Chem Lett. 2024 Apr 24;15(17):4587–92. doi: 10.1021/acs.jpclett.4c00768 (PMC11071072; doi:10.1021/acs.jpclett.4c00768)
Supplement: Supplementary file 1 — jz4c00768_si_001.pdf [file jz4c00768_si_001.pdf]

Supplementary Information: Preparation of Tautomer-Pure Molecular Beams by Electrostatic Deflection

Grite L. Abma,[†] Michael A. Parkes,[‡] and Daniel A. Horke[*,†]

†*Radboud University, Institute for Molecules and Materials, Heyendaalseweg 135, 6525 AJ Nijmegen, The Netherlands*

‡*Department of Chemistry, University College London, 20 Gordon Street, WC1H 0AJ London, United Kingdom*

E-mail: d.horke@science.ru.nl

1 Technical description of molecular beam apparatus

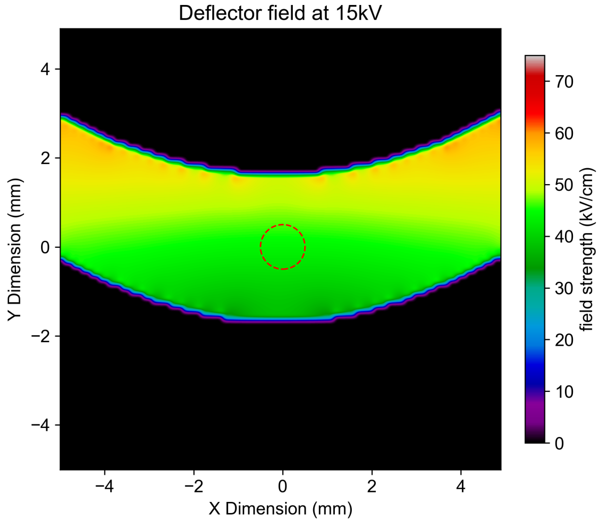

Figure S1: Cross-section of the electrostatic deflector, and indication of the electric field inside for an applied potential difference of 15 kV. The dashed circle indicates where the molecular beam enters the deflector.

Solid 2-pyridone (97% purity, Sigma-Aldrich) was used without further purification and placed inside the heated sample compartment of a pulsed Even Lavie Valve,[1] operated at 300 Hz, a valve temperature of 140 °C and a backing pressure of 80 bar of helium. The resulting molecular beam was first skimmed 117 mm downstream from the nozzle with a 2 mm diameter skimmer (all skimmers from Beam Dynamics Inc.), and again 248 mm from the nozzle with a 1 mm diameter skimmer. The produced molecular packet enters the 30 cm long electrostatic deflector 327 mm downstream of the nozzle. The used deflector is a rod-and-trough type ('a-type') electrostatic deflector.[2] A cross-section of this deflector, indicating the typical electric fields inside, is shown in Figure S1. The deflector is operated at a potential difference of 19 kV, corresponding to a maximum field gradient of about 60 kV/cm. After the deflector the molecular beam is skimmed once more 653 mm downstream of the nozzle with a 1.5 mm diameter skimmer.

The molecular beam is probed at the center of the VMI spectrometer,[3] which extracts ions parallel to the molecular beam and perpendicular to the laser propagation direction. The VMI spectrometer consists of 3 stainless steel plates (130 mm outer diameter, 2 mm thickness) placed 25 mm apart. All electrodes have central apertures of 30 mm diameter. The molecules were ionized using non resonant ionization by the frequency doubled output of a Ti:Sapph laser with a pulse duration of about 100 fs. The laser was attenuated to 30 µJ per pulse, operating at 600 Hz. The laser was focused using an f= 200 mm lens to a spot size of approximately 20 μm inside the center of the three-plate velocity-map imaging spectrometer, operated in time of flight mode for the deflection measurements and in VMI mode for imaging. The created ions or electrons are accelerated onto a dual micro-channel plate (MCP) detector (Photonis Inc., 40 mm diameter, 5 µm pore size), that is equipped with a P43 phosphor screen. Ion signal is read out from the back MCP and fed into a constant-fraction discriminator (Surface Concept) and then into a time-to-digital converter (Cronologic TimeTagger4) for analysis. The electrons are imaged using a CCD camera (Basler acA720-520um) at 600 Hz.

The photoelectron images are collected alternating between signal and background by running the molecular beam valve at half the repetition rate of the laser. These frames are sorted during analysis and used for a background subtraction. The collected photoelectron image is Abel-inverted using the PyAbel package,[4] in particular with an rbasex transform and Tikhonov regularization using the first order difference operator with a strength of 500.[5] The VMI spectrum was calibrated using the well-known photoelectron spectrum of Xenon.

Experimental deflection measurements were complemented by trajectory simulations. For these the Stark effect of both isomers was calculated using the freely available CMIstark software package.[6] Trajectories of individual quantum states ($J = 0 - 15$, 2000 trajectories per state) were then propagated through the experimental setup. Individual trajectories were combined to a simulated deflection profile through Boltzmann weighting, with the rotational temperature and the tautomer distribution fitted to the experimental data.[7] We fitted the result of the trajectory simulations to the experimental data by varying the rotational

temperature of the molecular beam and the isomer distribution, as described in section IV of this supplement.

2 Torsional barrier

The torsional barrier for rotation of the OH group in 2-hydroxypridine was calculated in Gaussian 16 with MP2 theory and a 6-311G++(3df, 3pd) basis by scanning the dihedral angle of the H atom on the OH group.[8] All other atoms were held fixed during this calculation. The results are shown in Figure S2 and indicate a barrier height of around 3200 cm^{-1}, and an energetic difference between the conformers of around 2000 cm^{-1}.

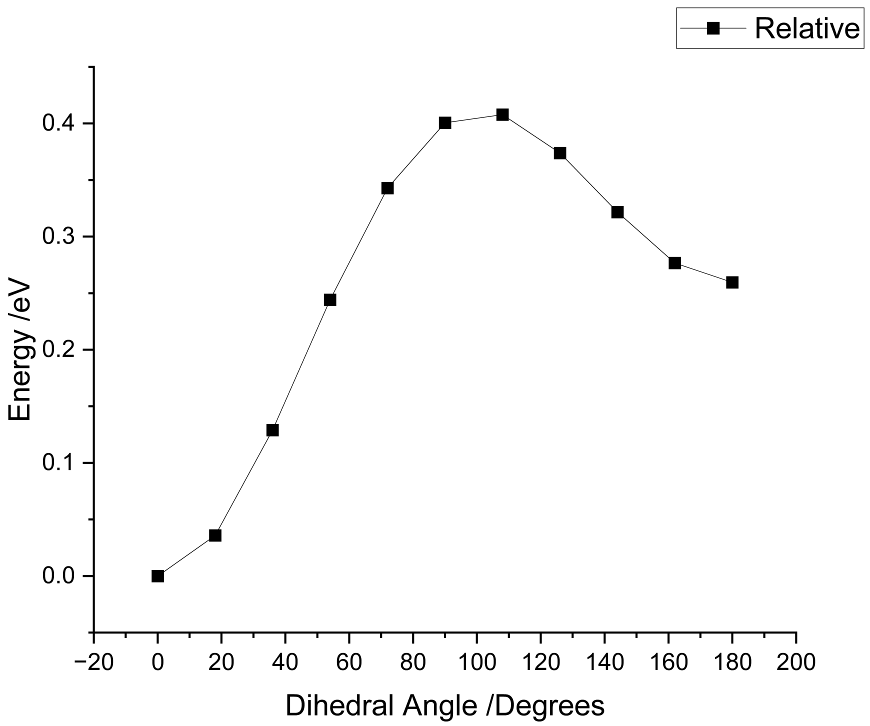

Figure S2: Calculated energies for a scan along the OH torsional mode.

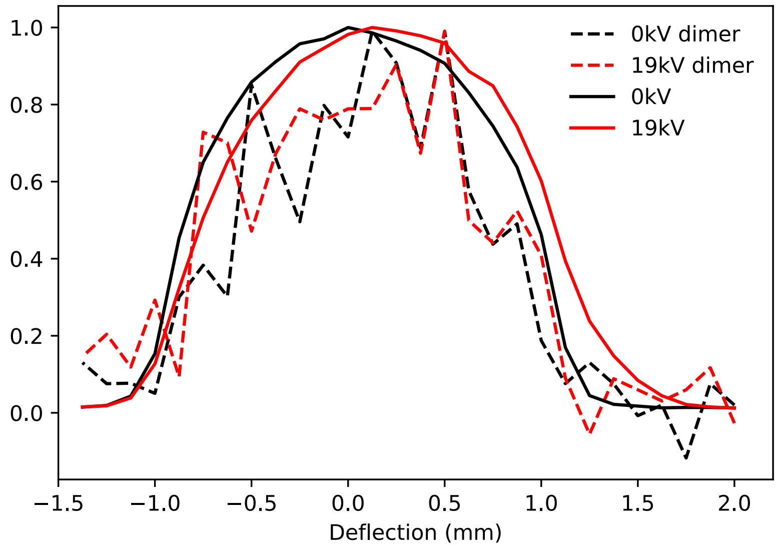

Figure S3: The deflection profile of the dimer (dashed lines) compared to the deflection profile of the monomer (solid lines). For both species the profile measured at 0 kV is shown in black and the profile measured at 19 kV is shown in red. No significant difference between the two profiles of the dimer can be seen.

3 Deflection of the dimer

In Figure S3 the recorded spatial density profiles of the dimer and monomer mass channels are shown. The monomer is shown as solid lines and the dimer as dashed lines, for both the beam profile measured at 0 kV is shown in black and the profile measured at 19 kV is shown in red. The spectra are normalized to maximum intensity. While significant deflection can be seen for the monomer, the dimer signal is quite noisy and no significant difference can be seen between the spectra measured at 0 kV and 19 kV. This confirms that the dimers present in the molecular beam do not deflect, and hence correspond to a homomeric dimer structure.

4 Fitting the trajectory simulations

We simulated 2000 trajectories for every quantum state of both tautomers of pyridone and matched these simulations to the experimental curve by first overlapping the experimental and simulated curves at 0 kV and then calculating the difference between the measurement and

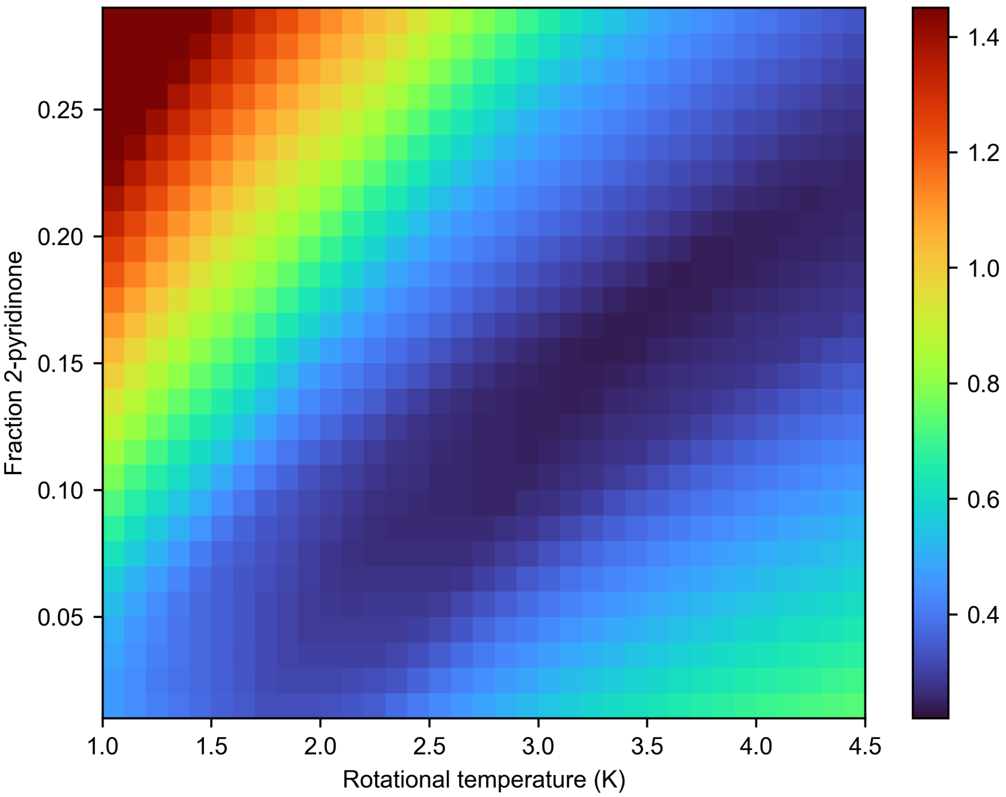

Figure S4: The summed difference between the measurement and result of trajectory simulations for a range of rotational temperatures and 2-pyridone fractions. Diagonal sections with similar error can be seen, indicating that lowering the 2-pyridone fraction or increasing the rotational temperature of the beam have very similar effects.

the simulation for the ten top points in the deflection measurements, so from 0.875 mm above the center to 2 mm above the center of the molecular beam. We calculated the differences for all rotational temperatures between 1 K and 4.5 K and 2-pyridone fractions between 0 and 0.3 (meaning 30% of the molecules in the beam are the 2-pyridone tautomer).

We summed the differences for all points to obtain a global error, which can be seen in Figure S4 as a 2D false color plot. The absolute magnitude of this error is not significant, and the figure clearly shows that there are diagonal sections with similar error. From this it can be concluded that decreasing the 2-pyridone fraction and increasing the rotational temperature have similar effects on the result of the simulations. The minimum global error was found for a rotational temperature of 3.4 K and a pyridone fraction of 0.16.

5 Optimized Structures

Table S1: Optimised Geometry of the Enol tautomer

	X /Å	Y /Å	Z /Å
C	0.898437611	0.02548441	0
C	0.186092878	1.230397375	0
C	-1.192533614	1.153116622	0
C	-1.811519638	-0.100368262	0
C	-1.00305688	-1.222419	0
N	0.336925773	-1.168652657	0
H	-1.78489808	2.058250311	0
H	0.717288413	2.169977928	0
H	-2.886629564	-0.199557088	0
H	-1.433218968	-2.215779351	0
O	2.245578148	0.081336102	0
H	2.553346479	-0.829182729	0

Table S2: Optimised Geometry of the Keto tautomer

	X /Å	Y /Å	Z /Å
C	1.058634553	0.054596935	-4.65436E-05
C	0.244010773	1.262877452	-5.0212E-06
C	-1.110471608	1.201643425	-3.7084E-06
C	-1.800831687	-0.054664498	-6.5051E-06
C	-1.056970927	-1.18396714	1.4958E-06
N	0.308568706	-1.118463644	-8.9457E-06
H	-1.685938621	2.118511224	5.7021E-06
H	0.778770606	2.200688113	1.42251E-05
H	-2.877234438	-0.10931574	-2.814E-07
H	-1.481526725	-2.176943354	1.51124E-05
O	2.274977728	0.00947336	2.57271E-05
H	0.854167668	-1.964766952	4.3992E-06

Table S3: Optimised Geometry of the Keto-Enol dimer

	X /Å	Y /Å	Z /Å
C	1.979044	-0.863604	0.002527
C	2.183294	1.549323	-0.001874
C	3.54958	1.499207	-0.003562
C	4.164028	0.227651	-0.002215
H	1.621169	2.472503	-0.002759
H	0.408859	0.451661	0.00204
H	4.12483	2.41147	-0.005873
H	5.244055	0.15464	-0.003558
O	1.223639	-1.85647	0.005158
H	3.864522	-1.898712	0.001692
C	-2.043306	-0.791914	-0.001346
C	-3.447702	-0.839516	-0.003795
C	-4.158153	0.344401	-0.002135
C	-3.465493	1.557979	0.001959
C	-2.083989	1.514524	0.004087
N	-1.377405	0.368465	0.00244
H	-5.239909	0.326918	-0.003941
H	-3.933913	-1.804179	-0.006855
H	-3.98112	2.50701	0.003436
H	-1.500849	2.428155	0.00729
O	-1.382371	-1.94025	-0.003018
H	-0.390037	-1.810418	0.000242
N	1.451498	0.410769	0.001137
C	3.41163	-0.918039	0.000708

Table S4: Optimised Geometry of the Keto-Keto dimer

	X /Å	Y /Å	Z /Å
C	1.838404	-0.833341	-0.035431
C	3.193671	-1.3082	0.001104
C	4.253741	-0.433369	0.025221
C	4.038553	0.965528	0.022671
H	3.339648	-2.379667	0.005877
H	5.264135	-0.820201	0.033138
H	4.855195	1.669214	0.034851
H	2.46856	2.460082	0.013416
C	-1.830297	0.826229	-0.00998
C	-3.18087	1.313172	-0.002167
C	-4.250862	0.450228	0.018691
C	-4.050888	-0.949894	0.028435
C	-2.760889	-1.413572	0.008942
N	-1.71438	-0.550348	-0.006802
H	-5.256998	0.849284	0.029458
H	-3.314852	2.385174	-0.013407
H	-4.875253	-1.644999	0.047787
H	-2.498141	-2.461758	0.018327
O	-0.808335	1.552795	-0.034814
N	1.707673	0.539895	-0.003877
C	2.743127	1.414591	0.015787
O	0.822712	-1.570866	-0.029791
H	-0.744527	-0.940461	-0.01559
H	0.732026	0.912846	-0.001899

References

(1) Even, U. Pulsed supersonic beams from high pressure source: simulation results and experimental measurements. *Adv. Chem.* **2014**, *2014*, 636042.

(2) de Nijs, A. J.; Bethlem, H. L. On deflection fields, weak-focusing and strong-focusing storage rings for polar molecules. *Phys. Chem. Chem. Phys.* **2011**, *13*, 19052–8.

(3) Eppink, A. T. J. B.; Parker, D. H. Velocity map imaging of ions and electrons using electrostatic lenses: Application in photoelectron and photofragment ion imaging of molecular oxygen. *Rev. Sci. Instrum.* **1997**, *68*, 3477–3484.

(4) Hickstein, D. D.; Gibson, S. T.; Yurchak, R.; Das, D. D.; Ryazanov, M. A direct comparison of high-speed methods for the numerical Abel transform. *Rev. Sci. Instrum.* **2019**, *90*, 065115.

(5) Gibson, S.; Hickstein, D.; Yurchak, R.; Ryazanov, M.; Das, D.; Shih, G. rBasex method from the PyAbel package. 2022; URL: `10.5281/zenodo.7438595`.

(6) Chang, Y.-P.; Filsinger, F.; Sartakov, B.; Küpper, J. CMIstark: Python package for the Stark-effect calculation and symmetry classification of linear, symmetric and asymmetric top wavefunctions in dc electric fields. *Comp. Phys. Comm.* **2014**, *185*, 339–349.

(7) Chang, Y.-P.; Horke, D. A.; Trippel, S.; Küpper, J. Spatially-controlled complex molecules and their applications. *Int. Rev. Phys. Chem.* **2015**, *34*, 557–590.

(8) Frisch, M. J.; Trucks, G. W.; Schlegel, H. B.; Scuseria, G. E.; Robb, M. A.; Cheeseman, J. R.; Scalmani, G.; Barone, V.; Petersson, G. A.; Nakatsuji, H. et al. Gaussian16 Revision C.01. 2016.
