# Supplementary material for: Preparation of Tautomer-Pure Molecular Beams by Electrostatic Deflection
Source: J Phys Chem Lett. 2024 Apr 24;15(17):4587–92. doi: 10.1021/acs.jpclett.4c00768 (PMC11071072; doi:10.1021/acs.jpclett.4c00768)
Supplement: Supplementary file 2 — jz4c00768_si_002.pdf [file jz4c00768_si_002.pdf]

jz-2024-00768v.R1

Name: Peer Review Information for "Preparation of Tautomer-Pure Molecular Beams by Electrostatic Deflection"

First Round of Reviewer Comments

Reviewer: 1

Comments to the Author

The work showcased here presents, for the first time, the separation of molecular tautomers within a gas-phase molecular beam, leading to a tautomererically pure gas-phase sample. Using the 2-hydroxypyridine/2-pyridone system, an initial molecular beam was passed through an electrostatic deflector which, though exploitation of the Stark effect, translated the keto 2-pyridone, on average, further than the 2-hydroxypyridine due to its larger dipole moment. This resulted in a region within the molecular beam where pure 2-pyridone was present. Such separation enabled the investigation of tautomer-specific photoelectron spectroscopy via multiphoton ionization, performed using a velocity-map imaging spectrometer. Extracted photoelectron spectra showed distinct differences in the combined and tautomer-pure regime.

While tautomer-specific dynamics have been resolved through use of high-resolution spectroscopy techniques before, this work has, for the first time, demonstrated the complete spatial separation of a tautomer within a molecular beam. This noteworthy advance gives rise to molecular samples that may be studied by a suite of techniques previously blind to tautomer specificity – and I anticipate this demonstration will serve as a catalyst for such work moving forwards. Overall, this paper was an enjoyable and interesting read. It demonstrates sufficient novelty and significance to warrant publication in The Journal of Physical Chemistry Letters. There are, however, some (mostly very minor) comments which should be addressed by the authors prior to publication:

1. Page 2 contains a small typographical error: “Studies of the relaxion dynamics where both tautomers absorb, ….”

2. On page 3, emphasis of the novelty of spatial separation of tautomers in a molecular beam is absent and I think the authors should sell their work more strongly here. I suggest the relevant paragraph start with ‘We show here, for the first time, spatial separation of tautomers in the gas phase…”. I also think the novelty of the work could be emphasized more strongly in the Abstract.

3. Remaining on page 3, the energetic barrier between tautomers is quoted as "34-38 kcal/mol". Is this value (or the stated unit) correct, as it seems rather high? Including a reference to back this up would be appropriate here. Furthermore, the unit switch to wavenumbers in the next sentence when mentioning the energetic difference between tautomers is unhelpful. To improve clarity, I recommend the barrier height is also expressed in wavenumber units.

4. On page 4, the first mention of the electrostatic approach mentions it "exploits the difference in the permanent dipole moment…". I think for complete clarity, this should be "electronic permanent dipole moment". This specificity is given later in this paragraph, and it should be given here also.

5. On page 4, reference to quantum calculations performed is made: "… and all spectral assignments are confirmed by quantum-chemical calculations…". Including the additional text "(see SI/ experimental methods)" at the end of this would be appropriate, to guide the reader to the calculation specifics.

6. Unless I've missed something, it seems that all simulations and modelling assume the minimum energy conformational structure for 2-hydroxypyridine, as shown in Fig. 1. Any free rotation of the OH group about the C-O bond will, however, presumably modify the dipole moment and hence impact on the simulations and modelling work. If the barrier to rotation is relatively large then it's not an issue, but if it's small could (or should) a dipole moment averaged over all rotation angles be used in place of that for the conformationally locked structure? Either way, the authors should quote (if known) or calculate this barrier and comment on it explicitly.

7. In the caption for Figure 3, there is no mention of the dotted black lines (relating to the deflection distances used in the subsequent fs-MPI experiment). There is also no mention that the data shown is for the m/z=95 channel exclusively, as stated in the main text. Finally, the legend has an error: "2-pyridinone" should be "2-pyridone".

8. On page 6, Figure 3 shows that at deflections of -1 mm or so the beam contains almost no 2-pyridone. Is there some technical (or other) reason why this region was not subsequently interrogated with VMI? This should be commented on as it would perhaps have been interesting/instructive to compare that data with the subtracted measurement in Fig. 4c.

9. On pages 8 and 9 the X state label attached to the cation ground state should (formally) have a tilde placed over it as we are dealing with a polyatomic molecule.

10. On page 9 when discussing the expected electron energies of the dimer systems, there is a typographical error: “(yielding expected electron kinetic energies ...”. Also, later on the same page “Both the features attributed to...”

11. On page 10, “custom-build” should be “custom-built”. Later in the same paragraph, I assume the 100 fs duration quoted is a full-width at half-maximum value? This should be explicitly stated.

12. In the supporting information, there is a typographical error: “The used deflector is a a rod-and-trough type”.

13. References contain both title-case and sentence-case. A uniform approach be better here.

14. In the SI, there are a couple of instances where “figure X” should be capitalized.

15. Finally, I think the TOC graphic provided is somewhat misleading as it implies that the left hand VMI data relates exclusively to 2-hydroxypyridine (when it doesn’t!). This should be reconsidered.

Reviewer: 2

Comments to the Author

Abma, Parkes and Horke present experimental results on electrostatic deflection of a molecular beam of 2-pyridone and 2-hydroxypyridine tautomers. By comparison to numerical simulations, they demonstrate that the more polar 2-pyridone tautomer (the keto form) can be separated from the 2-hydroxypyridine tautomer (the enol form) – and from dimers of the tautomers.

As a prototype demonstration of the use of a tautomer-selected sample, the authors record the photoelectrons, produced through three-photon ionization by an intense fs laser pulse. The two-dimensional electron images and the resulting kinetic energy distribution of the tautomer-selected sample differs significantly from those of the mixed sample, i.e. from the undeflected molecular beam.

Although, as the authors write themselves, electrostatic deflection is an established technique to separate different conformers of a molecule or molecules in different rotational states, the present work is the first to demonstrate the effect for a pair of tautomers. Given the important role played by tautomerism in notably organic chemistry and in biochemistry, I believe the results of the paper are important. In addition, the paper is well-written and easy to read – also for researchers that are not experts in manipulation of molecules with electrostatic fields.

As such, I recommend publication in JPC. I have no problem seeing the manuscript being published as a letter although I am not hundred percent sure that present significant new physical insights and/or extremely important results. I will leave the decision of a letter or a regular article to the editor subject to the argumentation from the authors about why a letter is the right format.

I have only a few minor points that the authors should address before acceptance of the manuscript:

1) Page 2, line 17: 'Here the extremely cold and collision'. Extremely is perhaps a bit of a strong description. In the current work the temperature is 3.4 K, which is actually not a particularly cold beam – and certainly not as cold as one can obtain e.g. by embedding molecules in a helium nanodroplet (0.37 K). I suggest to delete 'extremely'

2) Page 2, last line: 'ultrafast' -> 'ultrashort'

3) Page 3, line 27: 'distribution' -> 'population ratio'

4) Page 6, line 34-35: 'tightly focused probe laser beam' is too qualitative. Please give the spotsize, like \omega_0, in micrometer.

5) Page 6, line 42-43: 'The photoelectron spectrum was extracted using Abel inversion' . This is too brief. Please expand a bit, i.e explain that the 3D velocity distribution is obtained by Abel inversion of the radial distribution of the image and then the kinetic energy distribution by an appropriate transformation of probability distributions. Would also be helpful to know how the VMI spectrometer is calibrated. Perhaps this can go into the supplemental material.

6) Page 10, line 39: Please give the peak intensity of the laser pulse.

Author's Response to Peer Review Comments:

We thank the reviewers for their careful reading of our manuscript, and their overall very positive review of our work. We here provide a point-by-point response to all comments raised. We have also provided a PDF of the paper and supporting information with all changes highlighted.

Reviewer: 1 Comments:

The work showcased here presents, for the first time, the separation of molecular tautomers within a gas-phase molecular beam, leading to a tautomererically pure gas-phase sample. Using the 2-hydroxypyridine/2-pyridone system, an initial molecular beam was passed through an electrostatic deflector which, though exploitation of the Stark effect, translated the keto 2-pyridone, on average, further than the 2-hydroxypyridine due to its larger dipole moment. This resulted in a region within the molecular beam where pure 2pyridone was present. Such separation enabled the investigation of tautomer-specific photoelectron spectroscopy via multiphoton ionization, performed using a velocity-map imaging spectrometer. Extracted photoelectron spectra showed distinct differences in the combined and tautomer-pure regime.

While tautomer-specific dynamics have been resolved through use of high-resolution spectroscopy techniques before, this work has, for the first time, demonstrated the complete spatial separation of a tautomer within a molecular beam. This noteworthy advance gives rise to molecular samples that may be studied by a suite of techniques previously blind to tautomer specificity – and I anticipate this demonstration will serve as a catalyst for such work moving forwards. Overall, this paper was an enjoyable and interesting read. It demonstrates sufficient novelty and significance to warrant publication in The Journal of Physical Chemistry Letters. There are, however, some (mostly very minor) comments which should be addressed by the authors prior to publication:

1. Page 2 contains a small typographical error: "Studies of the relaxion dynamics where both tautomers absorb, ..." Reply: Done.

2. On page 3, emphasis of the novelty of spatial separation of tautomers in a molecular beam is absent and I think the authors should sell their work more strongly here. I suggest the relevant paragraph start with 'We show here, for the first time, spatial separation of tautomers in the gas phase...". I also think the novelty of the work could be emphasized more strongly in the Abstract.
Reply: We thank the review for this comment, and have slightly revised the abstract and introduction to highlight the novelty.

3. Remaining on page 3, the energetic barrier between tautomers is quoted as "34-38 kcal/mol". Is this value (or the stated unit) correct, as it seems rather high? Including a reference to back this up would be appropriate here. Furthermore, the unit switch to wavenumbers in the next sentence when mentioning the energetic difference between tautomers is unhelpful. To improve clarity, I recommend the barrier height is also expressed in wavenumber units.
Reply: We have changed the barrier height to wavenumber units, and included a reference.

4. On page 4, the first mention of the electrostatic approach mentions it "exploits the difference in the permanent dipole moment…". I think for complete clarity, this should be "electronic permanent dipole moment". This specificity is given later in this paragraph, and it should be given here also.
Reply: Done.

5. On page 4, reference to quantum calculations performed is made: "… and all spectral assignments are confirmed by quantum-chemical calculations…". Including the additional text "(see SI/ experimental methods)" at the end of this would be appropriate, to guide the reader to the calculation specifics.
Reply: Done.

6. Unless I've missed something, it seems that all simulations and modelling assume the minimum energy conformational structure for 2-hydroxypyridine, as shown in Fig. 1. Any free rotation of the OH group about the C-O bond will, however, presumably modify the dipole moment and hence impact on the simulations and modelling work. If the barrier to rotation is relatively large then it's not an issue, but if it's small could (or should) a dipole moment averaged over all rotation angles be used in place of that for the conformationally locked structure? Either way, the authors should quote (if known) or calculate this barrier and comment on it explicitly.
Reply: We have calculated the barrier to rotation of the OH group, and this is around 0.4 eV (~3200cm^{-1}), and hence much higher than the available energy. We have furthermore added a reference from previous microwave spectroscopy measurements that also did not observe the other conformation of 2-hydroxypyridine. We have added this information to the manuscript, with further details in the supplementary.

7. In the caption for Figure 3, there is no mention of the dotted black lines (relating to the deflection distances used in the subsequent fs-MPI experiment). There is also no mention that the data shown is for the m/z=95 channel exclusively, as stated in the main text. Finally, the legend has an error: "2-pyridinone" should be "2-pyridone". Reply: We have updated the legend accordingly.

8. On page 6, Figure 3 shows that at deflections of -1 mm or so the beam contains almost no 2-pyridone. Is there some technical (or other) reason why this region was not subsequently interrogated with VMI? This should be commented on as it would perhaps have been interesting/instructive to compare that data with the subtracted measurement in Fig. 4c.

Reply: We agree with the reviewer that this could have been interesting. However, the least deflected edge primarily contains molecules in high rotational states, complicating the comparison to deflected edge (or even undeflected) data.

9. On pages 8 and 9 the X state label attached to the cation ground state should (formally) have a tilde placed over it as we are dealing with a polyatomic molecule. Reply: Done.

10. On page 9 when discussing the expected electron energies of the dimer systems, there is a typographical error: "(yielding expected electron kinetic energies ...". Also, later on the same page "Both the features attributed to..." Reply: Done.

11. On page 10, "custom-build" should be "custom-built". Later in the same paragraph, I assume the 100 fs duration quoted is a full-width at half-maximum value? This should be explicitly stated.
Reply: Indeed the 100fs was referring to FWHM, this has been included in the manuscript now.

12. In the supporting information, there is a typographical error: "The used deflector is a a rod-and-trough type".
Reply: Done.

13. References contain both title-case and sentence-case. A uniform approach be better here.
Reply: We have formatted all references uniformly.

14. In the SI, there are a couple of instances where "figure X" should be capitalized. Reply: Done.

15. Finally, I think the TOC graphic provided is somewhat misleading as it implies that the left hand VMI data relates exclusively to 2-hydroxypyridine (when it doesn't!). This should be reconsidered.
Reply: We have provided a new TOC graphic that avoids this confusion.

Reviewer: 2

Comments:

Abma, Parkes and Horke present experimental results on electrostatic deflection of a molecular beam of 2-pyridone and 2-hydroxypyridine tautomers. By comparison to numerical simulations, they demonstrate that the more polar 2-pyridone tautomer (the keto form) can be separated from the 2-hydroxypyridine tautomer (the enol form) – and from dimers of the tautomers.

As a prototype demonstration of the use of a tautomer-selected sample, the authors record the photoelectrons, produced through three-photon ionization by an intense fs laser pulse. The two-dimensional electron images and the resulting kinetic energy distribution of the tautomer-selected sample differs significantly from those of the mixed sample, i.e. from the undeflected molecular beam.

Although, as the authors write themselves, electrostatic deflection is an established technique to separate different conformers of a molecule or molecules in different rotational states, the present work is the first to demonstrate the effect for a pair of tautomers. Given the important role played by tautomerism in notably organic chemistry and in biochemistry, I believe the results of the paper are important. In addition, the paper is well-written and easy to read – also for researchers that are not experts in manipulation of molecules with electrostatic fields.

As such, I recommend publication in JPC. I have no problem seeing the manuscript being published as a letter although I am not hundred percent sure that present significant new physical insights and/or extremely important results. I will leave the decision of a letter or a regular article to the editor subject to the argumentation from the authors about why a letter is the right format.

I have only a few minor points that the authors should address before acceptance of the manuscript:

1) Page 2, line 17: 'Here the extremely cold and collision'. Extremely is perhaps a bit of a strong description. In the current work the temperature is 3.4 K, which is actually not a particularly cold beam – and certainly not as cold as one can obtain e.g. by embedding molecules in a helium nanodroplet (0.37 K). I suggest to delete 'extremely' Reply: Done.

2) Page 2, last line: 'ultrafast' -> 'ultrashort' Reply: Done.

3) Page 3, line 27: 'distribution' -> 'population ratio' Reply: Done.

4) Page 6, line 34-35: 'tightly focused probe laser beam' is too qualitative. Please give the spotsize, like \omega_0, in micrometer.
Reply: This information is already included in the SI, and the reader is referred to this in the main manuscript.

5) Page 6, line 42-43: 'The photoelectron spectrum was extracted using Abel inversion' . This is too brief. Please expand a bit, i.e explain that the 3D velocity distribution is obtained by Abel inversion of the radial distribution of the image and then the kinetic energy distribution by an appropriate transformation of probability distributions. Would also be helpful to know how the VMI spectrometer is calibrated. Perhaps this can go into the supplemental material.
Reply: Additional information on the Abel inversion is already included in the supporting information. We have added a statement to the main manuscript referring the reader here. We have added details of the calibration of the VMI spectrometer to the supporting information.

6) Page 10, line 39: Please give the peak intensity of the laser pulse. Reply: Done.
